# Paediatric Outpatient Parenteral Antimicrobial Therapy (OPAT): An e-survey of the experiences of parents and clinicians

**DOI:** 10.1371/journal.pone.0249514

**Published:** 2021-04-02

**Authors:** Bernie Carter, Debra Fisher-Smith, David Porter, Steven Lane, Matthew Peak, David Taylor-Robinson, Louise Bracken, Enitan D. Carrol

**Affiliations:** 1 Faculty of Health, Social Care and Medicine, Edge Hill University, Ormskirk, United Kingdom; 2 Alder Hey Children’s NHS Foundation Trust, Liverpool, United Kingdom; 3 Department of Clinical Infection, Microbiology and Immunology, University of Liverpool, Liverpool, United Kingdom; 4 Department of Infectious Diseases and Immunology, Alder Hey Children’s NHS Foundation Trust, Liverpool, United Kingdom; 5 Institute of Translational Medicine, University of Liverpool, Liverpool, United Kingdom; 6 Paediatric Medicines Research Unit, Institute in the Park, Alder Hey Children’s NHS Foundation Trust, Liverpool, United Kingdom; 7 Department of Public Health and Policy, University of Liverpool, Liverpool, United Kingdom; 8 Department of Clinical Infection, Microbiology and Immunology, Institute of Infection and Global Health, University of Liverpool, Liverpool, United Kingdom; 9 Liverpool Health Partners, Liverpool Science Park, Liverpool, United Kingdom; International Medical University, MALAYSIA

## Abstract

**Background:**

Little evidence exists about parental satisfaction and their influence on referral to paediatric Outpatient Parenteral Antimicrobial Therapy (OPAT).

**Aim:**

This study aimed to examine the experiences of parents, children and clinicians of OPAT at a large tertiary children’s hospital.

**Method:**

A prospective e-survey, using closed and open questions, of parents (n = 33) of 33 children who had received OPAT (3 children completed a survey), and clinicians (n = 31) involved in OPAT at a tertiary hospital. Data were collected September 2016 to July 2018.

**Results:**

Data were analysed using simple descriptive statistics. The results show that OPAT offered benefits (less stress, re-establishment of family life) compared to hospital-based treatment for parents and children, although some were anxious. Clinicians’ referral judgements were based on child, home, and clinical factors. Some clinicians found the process of referral complex.

**Conclusion:**

Most parents and children were satisfied with the OPAT service and preferred the option of home-based treatment as it promoted the child’s comfort and recovery and supported family routines.

## Introduction

In a consensus statement on good practice recommendations for paediatric Outpatient Parenteral Antimicrobial Therapy (OPAT) in the UK, OPAT for children is defined as the parenteral administration of antimicrobials for at least two consecutive days without an intervening hospitalisation [1]. OPAT services mean that hospital admission is either avoided altogether or patients are discharged earlier than would otherwise be possible. Outpatient treatment of children with serious bacterial infections (SBIs) using parenteral antimicrobial therapy commenced in the mid-1970s [2] and the scope, context and number of services has increased and children with diverse needs and conditions and across the age span now receive OPAT [3–5]. This partly reflects a turn to more home-based and family-centred care across health services [6]. It also a reflects other policies such as reducing the length and number of inpatient stays, a belief that OPAT is a more cost-effective option when compared to continued inpatient care [7–9] and it may reduce healthcare-associated infections [1]. However, OPAT is not without risk [10] and a systematic review concludes further improvements in paediatric OPAT are needed for safe and effective implementation [4]. Additionally, few studies have reported in any detail about the OPAT experiences of children and their parents. Most studies that do exist typically have quite a narrow focus on overall satisfaction [11] rather than exploring wider facets of experience. The most-detailed evidence on patient experiences comes from two qualitative studies of adult patients receiving OPAT [12, 13] and from an in-depth qualitative study of parents whose children had received OPAT [14]. The findings from these three studies reflect that despite the benefits of OPAT, some aspects can be frustrating for patients and parents.

This paper reports on Phase 1 (survey phase) of a larger study. The aim of this phase of the study was to examine the experiences of parents, children and clinicians of the OPAT service.

## Materials and methods

### Study design

A prospective e-survey, using closed and open questions.

### Participants and setting

Children aged 0–16 years and their parents who had been referred to the OPAT service based at Alder Hey Children’s Hospital, a tertiary children’s setting in Liverpool in the UK were invited to participate in the study.

Children were enrolled from September 2016 to July 2018. All patients referred for admission to OPAT are reviewed. There are no upper or lower age limits, all infection-related diagnoses are accepted, and the duration of treatment can be from a few hours to weeks or months. Children are not accepted if there is significant concern for the safety of the child or nursing staff visiting the child’s home, if the child is judged not well enough to be cared for safely at home, or if the proposed treatment is felt to be inappropriate by the OPAT team.

Following completion of their OPAT care, all parents (n = 56) and children (if appropriate) who gave written consent or assent to be part of the main study were sent a link to the e-survey by their preferred method of contact (email or text). Parents and/or children were also given the opportunity to complete the survey by telephone.

Clinicians, (n = 78), identified as being involved in OPAT either directly (e.g., member of the OPAT team) or indirectly (e.g., referred child for OPAT), were sent a link to the e-survey via their official email address. The aim was to gain the views of a cross-section of clinicians involved with OPAT.

### E-surveys

The e-surveys (non-validated) were designed and refined as a result of engagement with parents and children to ensure that the survey adequately explored the range of experiences of the OPAT service and how OPAT influenced the lives of the child and the family. Specific e-surveys were developed for children/young people (aged 6–16 years), parents and clinicians.

The surveys used a mix of primarily closed questions with some open-ended questions. The Parent Survey consisted of 18 main questions across two main domains: ‘Background Information on Child and Parent’ (including child’s age, reason for treatment, number of siblings living at home; everyday work and support situation); and ‘Experience with OPAT’ (including where in the home the treatment was given, comparison between hospital and home-based treatment, parent’s own and their perception of child’s satisfaction including support, impact, worries, concerns, best and worst things about OPAT). There was a final question about parent’s educational attainment to allow assessment of socioeconomic status.

The Child Survey was simpler and more concise asking children to agree or disagree with core statements about OPAT such as ’I would have preferred to stay in hospital for my medicine’, ’I felt OK about having my infection medicine at home’. They were also asked about the best things about OPAT and the things they would like to change.

The Clinician Survey consisted of four domains: Background Information (role, setting); Knowledge of Referral Process (number of referrals, ease of referral, time burden, satisfaction with process); Factors affecting referral of children (clinical, parent-oriented, home-oriented factors); and Experience of Service (comparability with hospital-based service in terms of administration, management of adverse effects).

Submission of the e-survey was taken as consent (or assent) to participate in the study. All e-survey responses were anonymous, unless they chose to share their contact details for potential participation in Phase 2 (interviews).

### Analysis

The data from the closed questions from the e-surveys were analysed using simple descriptive statistics using Excel. The qualitative responses from the open-ended questions were considered in relation to the closed-responses and used to illustrate the participants’ experiences and perspectives.

### Ethics

Ethics approval was gained via the NW Greater Manchester West Research Ethics Committee (16/NW/0440). All relevant governance protocols relating to data management and pseudo-anonymisation were followed. Written consent was supplied by the parent/guardian of the patient giving consent for data collected during the study to be accessed and analysed by individuals for the purposes of research.

## Results

The results are reported separately for the parents and clinicians. Since not all respondents answered every question or each item in a multi-item question we have chosen to report raw numbers. In the quotations taken from the open-ended questions to illustrate the results, the following initials are linked to quotes: M (mother); F (father); C (child); and Clin (clinicians).

### Demographics of parents and children

Thirty three parents (32 mothers and 1 father) of 33 children participated and completed e-surveys (58% response rate); 3 children (2 boys, 1 girl; aged 6–9 years) assented and submitted an e-survey (Table 1).

**Table 1:** Summary data for all children: Age, total days of OPAT and total IV duration.

	Age (weeks/yrs/months)	Total days OPAT	Total IV duration
Mean	5yr 2m	9.50	13.50
Min	4 weeks	1.00	2.00
Max	15yr 4m	35.00	56.00
Median (IQR 25-75th)	3 yr 9m (0.8–7.8)	7 (4.8–12.3)	10.5 (7–14.3)

The children (male n = 17, female n = 16); were aged between 4 weeks to 15 years (mean 5.2 years). The duration of OPAT ranged from 1–35 days (mean 9.5 days; median 7.0 days). Most of the children (n = 30) had no previous experience of OPAT and most had been hospitalised prior to being referred to OPAT (n = 30). The children were receiving treatment for a variety of conditions: the top three conditions reported by parents were lower respiratory tract infection (n = 6), bloodstream infection (n = 5), and meningitis (n = 4) (see Table 2).

**Table 2:** Parent-reported reasons for OPAT (may not be clinically accurate).

Reason	Details
Respiratory	N = 6. Chest infection acute (n = 3); Chest infection chronic (n = 3)
Bloodstream	N = 5. Strep A (n = 2); Staph A (n = 1); E.Coli (n = 1); not stated (n = 1)
Meningitis	N = 4. Bacterial (n = 1); Meningococcal (n = 1); Suspected meningitis (n = 2)
Abdominopelvic	N = 2. Ruptured appendix (n = 1); Cholangitis (n = 1)
Genitourinary	N = 4. Kidney infection
Ear, Nose and Throat	N = 3. Sinus infection (n = 1); Tonsillitis, cervical lymphadenitis (n = 1) Mastoiditis (n = 1)
Musculo-skeletal	N = 1. Osteomyelitis (n = 1);
Line-related	N = 2. Broviac (n = 1); Central line (n = 1)
Skin and soft tissue	N = 1. Ocular cellulitis (n = 1)
Other/description unclear	N = 5. Infection, type not stated (n = 2); Prophylactic cover, post-operatively (n = 1); Pain, excruciating (n = 1); Save his life (n = 1)

In nine families, the child on OPAT was the only child living at home; 17 families had one other child living at home with six families having two other and one family have three other children living at home. Twenty-two parents worked out of the house either full-time (n = 12) or part-time (n = 10) out with the remaining 11 parents working as a full-time mother or father in the home. Most of the parents (n = 24) reported having a lot of support they could call on although six parents reported having little support and having to manage on their own.

### Demographics of clinicians

Thirty-one clinicians returned surveys (40% response rate). Of these 16 were doctors (consultants, n = 12; registrars n = 3; junior doctor n = 1); 14 were nurses (nurse specialist n = 4; advanced nurse specialist n = 4; sister/charge nurse n = 3; staff nurse n = 3); and one respondent stated ‘other’. Most worked within the tertiary paediatric setting (n = 29), the remaining two worked in either an acute children’s setting or a community setting. Most of the clinicians (n = 17) reported that they worked in general paediatrics; the others worked in neurology (n = 5); emergency department (n = 3), oncology (n = 3), cystic fibrosis (n = 2); and ‘other’ (n = 1).

Of the 20 clinicians who reported they had referred children to the OPAT service, 12 had referred between 1 and 10 children, 5 had referred 11–20 children and only 3 had referred more than 20 children.

### Parent and child

Of the 30 parents who responded to this question, most (n = 28) were quite or very satisfied with the OPAT service; with only 2 parents expressing dissatisfaction.

**Fig 1:**
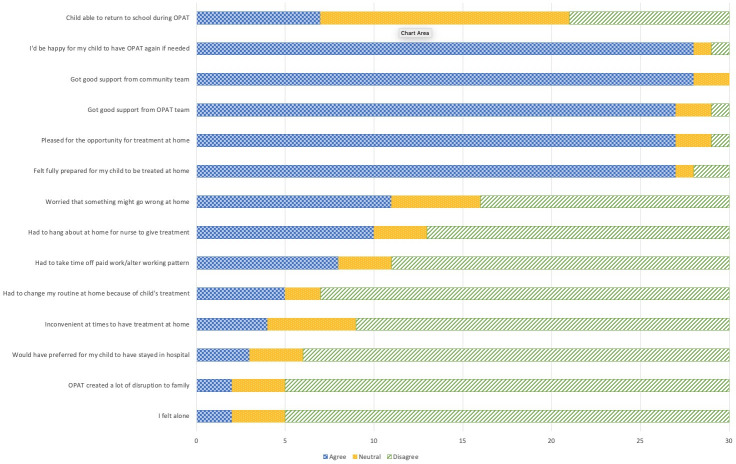
Extent of agreement and disagreement with statements about parents’ experiences and thoughts of the service (n = 30).

When questioned in more detail about aspects of their experience, parents again tended to respond positively (see Fig 1) even if they had never expected that their child could be treated at home. One mother for example explained:

*“Having my son’s treatment at home was something I never knew existed*. *I really think this is such a great way to free up hospital beds and allowed my child to get back into his normal routine, and he was able to attend school” (M)*.

The children were glad to be home because it was more *“comfortable”*, they were able to *“sleep in my own bed”* and *“didn’t have to have nurses checking me and coming in and out of my room all day and night”*.

Most parents agreed that they received good support from the community team (n = 28) and OPAT team (n = 27) and most (n = 27) were pleased to have been given the opportunity to have the treatment at home, although 3 parents would have preferred for their child to stay in hospital. The majority (n = 27) reported they were fully prepared for their child to have treatment at home. All three children reported that the nurses were *“kind”*.

Most parents (n = 28) reported being happy for their child to have OPAT again, if needed and there being no major disruption to family life (n = 25) with only 5 parents reporting that routine at home had to change. However, any disruption was seen in the context of higher levels of disruption that occurred during the child’s hospitalisation with one mother explaining “*Having the treatment at home still disrupted me working but I couldn’t work at all while she was in hospital”* (M). However, 10 parents and all three children reported having to wait around at home during the 2-hour visit window for the nurse to arrive, although this was not seen as unduly problematic.

Although there were no significant negative responses, there was a mixed response to some of the statements. Whereas, 19 parents were ether unworried or neutral about something going wrong, 11 parents were worried. One mother was noted that she was *“worried about the amount of medication given at one time”* compared to the split doses given in hospital. Others talked of initial worries about *"being forgotten"* by the OPAT nurses or were worried that their child was *"still a bit ill"*.

Parents were generally positive about their child’s experience of OPAT, and most (n = 19) agreed that their child was *"OK"* having the treatment at home and 15 were pleased to have the opportunity to be given the treatment at home. Parents also agreed that the community team (n = 17) or the OPAT service (n = 17) supported their children; typically, the nurses were described as being *“very caring*, *polite and professional”* and supportive as they *“answered any questions that we had”* (M). The nurses were supportive and flexible in relation to administering the medication, as one mother explained:

*The team were fantastic and tried to make the experience as easy as possible including doing the morning treatment in school which was a massive help to me and my daughter*. *(M)*.

Parents reported that their child continued to be able to join in family activities (n = 17) and that their child would be happy to have the treatment at home in the future (n = 18). Not all children were old enough or well enough to return to school, although 7 parents reported that their child returned to school.

Although four parents reported that their child was upset when they had their treatment, most children (n = 18) were not upset. Only two parents thought their child would have preferred to have stayed in hospital for treatment.

The majority of parents and all three children considered that having antibiotic treatment at home rather than hospital was better (n = 22 of 30), with 5 parents being neutral and only 3 reporting it to be worse. One OPAT-experienced mother explained: *"we have used OPAT in the past before this time*, *and it works well for my family"* (M).

The children identified areas that they thought could be improved in the services including consistency in the nurse who gave the treatment, *“I would of preferred the same nurse because I liked the first nurse a lot and I had a different nurse each time”*, being prepared for how many nurses came to their home, *“sometimes it was one and sometimes it was two”*. One child felt the medication should be *“already made up”* because:

*“it takes a long time to make up the medicine and put it in the syringe*. *The big needles scared me” (C)*.

### Clinicians

**Fig 2:**
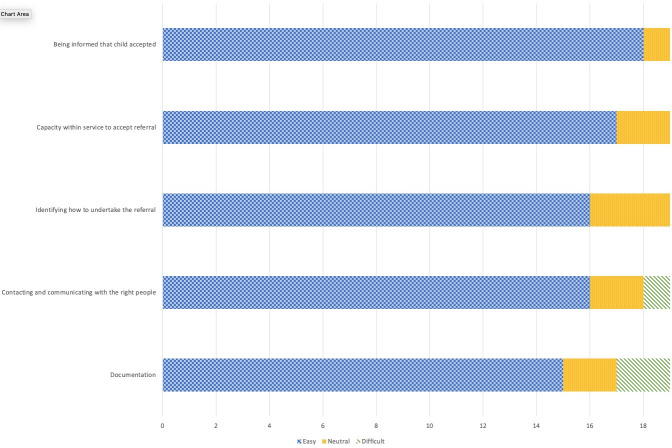
Reported ease of key aspects of the OPAT referral process for clinicians.

Overall, clinicians’ responses were positive about the OPAT service with most reporting that the key aspects of the OPAT service were ‘easy’ (see Fig 2). When asked to rate which aspects of the OPAT referral process were easy/dififcult, most clinicians found the processes to be easy, although two reported that OPAT documentation was difficult, with one stating *“Documentation for prescribing is very complicated and not user friendly”*. Connecting with the *“right people”* was described by some as difficult with one report of being *“pushed from pillar to post”*. The process of referral was seen as time-consuming by some clinicians who noted that it *“takes clinicians away from acute ED work* …*to organise”*. A *“more streamlined path”* with a *“single point of contact that is consistent regardless of time of day”* were suggestions for improving the process of referral. Another comment indicated that the service might be under-used with, for example, *“must be more patients we can refer but no visible reminders on a day-to-day basis” (Clin)*.

**Fig 3:**
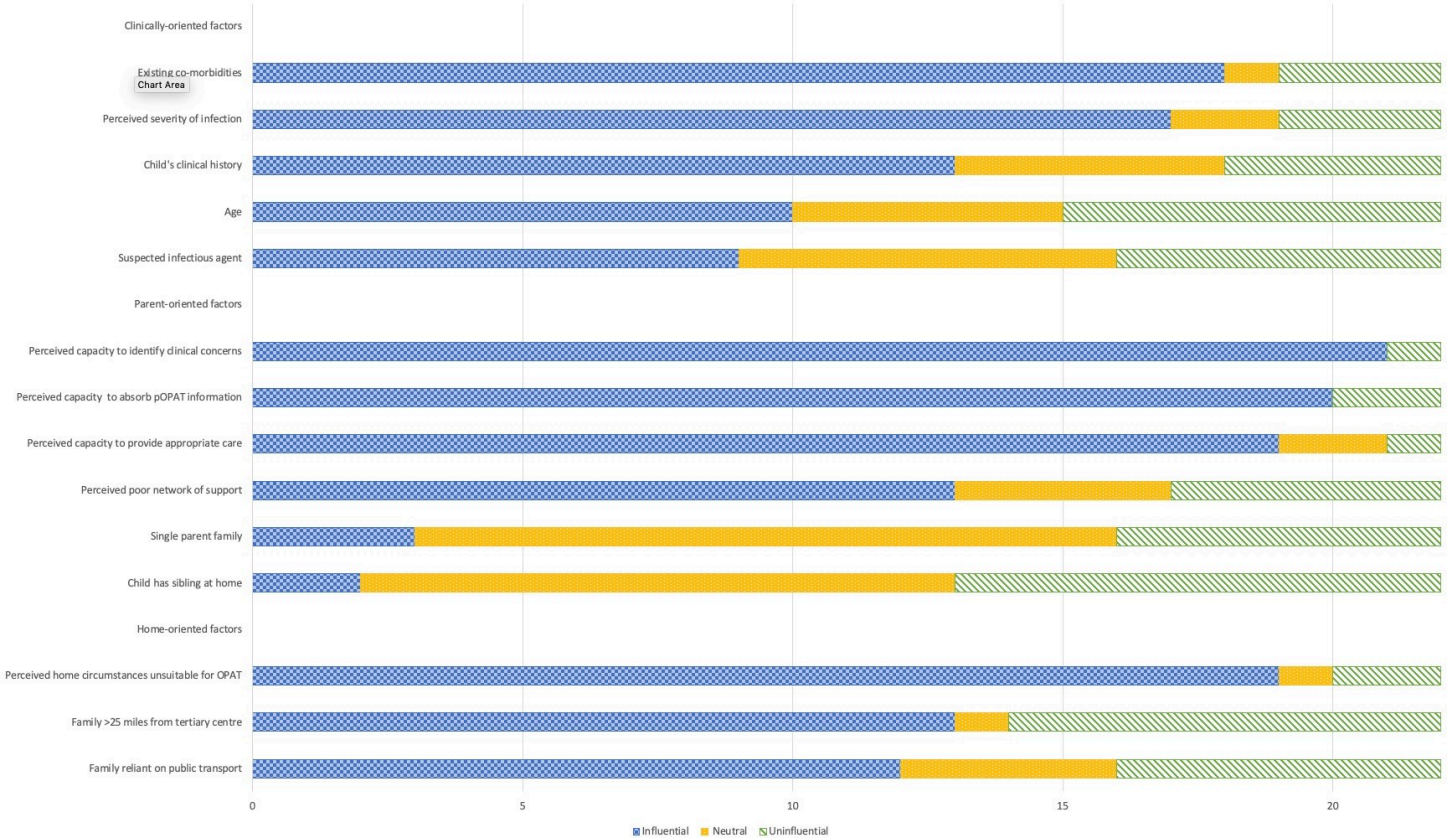
Clinical, parent-oriented and home-oriented factors influencing clinicians’ decisions not to refer a child to the OPAT service.

A child’s existing co-morbidities was the clinical factor that most clinicians (n = 18) reported would influence their decision not to refer a child to the OPAT service (see Fig 3). The perceived severity of the infection was the next most influential factor for 17 clinicians. The child’s clinical history was also reported as being influential (n = 13) although 5 clinicians considered this factor as neither influential nor uninfluential. In relation to the age of the child, 10 clinicians indicated that this was an influencing factor. There was less consensus about the suspected infectious agent.

Overall, various aspects of their perception of a parental capacity were the most influential parent-orientated factors that would sway a clinician’s decision not to refer a child to OPAT (see Fig 3). These factors were perceived capacity of parents to identify clinical concerns (n = 21); absorb OPAT-related information (n = 20) and provide appropriate care (n = 19). The perception that a parent had a poor network of support was influential for 13 clinicians, although 5 considered this to be uninfluential. Few clinicians reported single parent family status (n = 3) or the presence of siblings at home (n = 2) as being influential.

The majority of clinicians (n = 19) reported that their perception of the home circumstances of the family was something they took into account when considering referral to OPAT. Other factors including distance from the tertiary centre (n = 13) and reliance on public transport (n = 12) were also seen as influential (see Fig 3).

Reasons why referrals to the OPAT service were declined included lack of provision of local community services or *“capacity of existing teams to deliver OPAT”*. Regimen-related issues included *“antibiotics needed too frequently”*, *“timing of drugs* ‥*not abiding by OPAT times”*, *“blood results not acceptable for required antibiotic”* and *“infusions rather than bolus injections needed”* (referring to some areas where the community teams do not have the staffing or equipment to give infusions).

## Discussion

Whilst there is a developing body of evidence addressing the clinical outcomes of children requiring OPAT [4, 10], it is also clear that there is a real deficit of evidence about the experiences of children and their parents [15] and almost no literature considering the perspectives of clinicians. This study addresses this deficit by exploring these perspectives. Despite some differing opinions, overall there was a strong sense of satisfaction with OPAT as it offered the benefits of being together as a family at home, reduced the stress [16] and disruptions associated with hospitalisation and created opportunities for the child (and family) to resume their usual routines. The reduction in the disruption to family life and routine are often reported as being key benefits arising from OPAT [1] and care at home [17].

Although antimicrobial treatment at home was generally preferred by the parents and children, a significant proportion of parents were worried that something might go wrong at home, although little detail was provided about how extensive these worries were. The evidence suggests that OPAT is safe [15]; however, reassurance from professionals about the safety of OPAT at the time of referral and ongoing support from the OPAT nurses, cannot necessarily allay these worries. Findings from the qualitative component of this study reveal that parents worried about aspects of care such as displacement of lines and concerns about the cleanliness of the home; other work reports parental anxiety related to OPAT [18]. Studies focusing on adult OPAT patients have noted their worries about potential hazards in the home and the strategies employed to mitigate them [19] and the value of care co-ordination packages in supporting good patient outcomes [20].

The children who responded would have liked more consistency in the nurse(s) who gave the treatment; this makes sense in that children can develop stronger, closer and more trusting relationships with a nurse who is familiar to them. However, the parents praised the nurses for their skills and support and this triadic relationship (child-nurse-parent) is essential to managing both child and parents’ concerns, as is evident in child-centred [21] and in family centred care [22, 23].

Our findings reveal that a child’s existing co-morbidities were the clinical factor that most clinicians reported would influence their decision not to refer a child to the OPAT service. Other studies have also acknowledged that the child’s condition is influential in their decision to refer to OPAT [24–26]. Other important factors that would sway a clinicians’ decision not to refer a child to OPAT were perceptions of parental capacity and to a child’s perceived home circumstances. These perceptions, and the resulting clinical judgement, reflect other studies have indicated that the ’stability’ of home circumstances is influential in decision-making [27]. A final factor was the perceived complexity of the referral process for clinicians who were not regular referrers; other studies have identified organisational barriers as a limiting factor to the use of OPAT [28]. These factors are important to acknowledge as they appear to influence clinical decision-making in relation to OPAT referral. Ensuring the safety and quality of OPAT service delivery is essential [29] and patient selection is a key element that requires consideration [30] but this needs to also acknowledge any unconscious bias against children from perceived poorer home circumstances.

A strength of our study is that both views of parents/carers as well as clinicians are captured. Additionally, as the service studied operates across primary, secondary and tertiary settings, we believe our results are generalisable to other services in the United Kingdom. One limitation of the study is that the responses were categorical or ordinal data, thus limiting the statistical analysis performed.

## Conclusions

Although anxiety was evident for some parents and children, most were satisfied with the OPAT service and preferred the option of home-based treatment as it promoted the child’s comfort and recovery and supported family routines. Some clinicians found the process of referral complex and thought it could be better streamlined and made more user-friendly. For clinicians, their decision to refer a child to the OPAT service was most influenced by child factors (the child’s existing co-morbidities); parent factors (perception of a parent’s capacity to cope at home) and home factors (including distance from the tertiary centre and reliance on public transport).
